# A Dose-Response Relationship between Organic Mercury Exposure from Thimerosal-Containing Vaccines and Neurodevelopmental Disorders

**DOI:** 10.3390/ijerph110909156

**Published:** 2014-09-05

**Authors:** David A. Geier, Brian S. Hooker, Janet K. Kern, Paul G. King, Lisa K. Sykes, Mark R. Geier

**Affiliations:** 1 Institute of Chronic Illnesses, Inc., 14 Redgate Ct., Silver Spring, MD 20905, USA; E-Mails: davidallengeier@comcast.net (D.A.G.); jkern@dfwair.net (J.K.K.); 2 Department of Biology, Simpson University, 2211 College View Dr., Redding, CA 96003, USA; E-Mail: bhooker@simpsonu.edu; 3 Department of Psychiatry, University of Texas Southwestern Medical Center at Dallas, 5353 Harry Hine Blvd., Dallas, TX 75390, USA; 4 CoMeD, Inc., 14 Redgate Ct., Silver Spring, MD 20905, USA; E-Mails: paulgkingphd@gmail.com (P.G.K.); syklone5@verizon.net (L.K.S.)

**Keywords:** attention deficit, autism, ethylmercury, merthiolate, thiomersal

## Abstract

A hypothesis testing case-control study evaluated concerns about the toxic effects of organic-mercury (Hg) exposure from thimerosal-containing (49.55% Hg by weight) vaccines on the risk of neurodevelopmental disorders (NDs). Automated medical records were examined to identify cases and controls enrolled from their date-of-birth (1991–2000) in the Vaccine Safety Datalink (VSD) project. ND cases were diagnosed with pervasive developmental disorder (PDD), specific developmental delay, tic disorder or hyperkinetic syndrome of childhood. In addition, putative non-thimerosal-related outcomes of febrile seizure, failure to thrive and cerebral degenerations were examined. The cumulative total dose of Hg exposure from thimerosal-containing hepatitis B vaccine (T-HBV) administered within the first six months of life was calculated. On a per microgram of organic-Hg basis, PDD (odds ratio (OR) = 1.054), specific developmental delay (OR = 1.035), tic disorder (OR = 1.034) and hyperkinetic syndrome of childhood (OR = 1.05) cases were significantly more likely than controls to receive increased organic-Hg exposure. By contrast, none of the non-thimerosal related outcomes were significantly more likely than the controls to have received increased organic-Hg exposure. Routine childhood vaccination may be an important public health tool to reduce infectious disease-associated morbidity/mortality, but the present study significantly associates organic-Hg exposure from T-HBV with an increased risk of an ND diagnosis.

## 1. Introduction

Thimerosal is an organic-mercury (Hg) compound (49.55% Hg by weight) added to vaccines as a preservative, typically at concentrations from 0.005% to 0.01% (12.5 µg organic-Hg or 25 µg organic-Hg per 0.5-mL vaccine dose) [1]. Thimerosal rapidly dissociates into ethyl-Hg chloride, ethyl-Hg hydroxide and sodium thiosalicylate in saline solutions [2], and the resulting ethyl-Hg species have high affinities for being bound to free thiols in living systems [3]. U.S. Infants receiving the recommended routine childhood vaccination schedule in the 1990s may have been exposed to bolus doses of Hg nominally ranging from 12.5 µg organic Hg to 62.5 µg organic-Hg that collectively added up to nominally 200 µg organic-Hg from thimerosal-containing vaccines (TCVs) during the first six months of life (>50% of all Hg exposure when considering environmental sources of Hg) [4].

A hypothesis testing case-control study was undertaken to evaluate the potential dose-response relationship between organic-Hg exposure from thimerosal-containing hepatitis B vaccines (T-HBVs) administered within the first six months of life and the subsequent risk of being diagnosed with specific neurodevelopmental disorders (NDs) within the Vaccine Safety Datalink (VSD) database.

## 2. Experimental Section

### 2.1. Institutional Review Board Approval

The study protocol employed was approved by the Centers for Disease Control and Prevention (CDC), the Institutional Review Board (IRB) of Kaiser Permanente North-West (KPNW) and the IRB of Kaiser Permanente Northern California (KPNC). The data were analyzed at the secure Research Data Center of the National Center for Health Statistics in Hyattsville, MD. The views expressed in this study do not necessarily reflect those of the CDC nor Kaiser Permanente.

The VSD project was created in 1991 by the National Immunization Program (NIP) of the CDC [5,6,7]. The project links medical event information, specific vaccine history and selected demographic information from the computerized databases of several Health Maintenance Organizations (HMOs). The cohort examined was comprised of individuals with non-missing date of birth and non-missing gender, who were HMO-enrolled from birth.

### 2.2. Determining the Population at Risk

A cohort of over 1.9 million infants enrolled in the VSD project (updated through the end of 2000) from KPNW, Kaiser Permanente Colorado (KPC) and KPNC was examined using SAS^®^ software.

### 2.3. Determining Cases

The outcome files (inpatient and outpatient diagnoses) from this population were reviewed to find the first instance of International Classification of Disease, 9th revision (ICD-9), diagnosed NDs by examining the following outcomes: pervasive developmental disorder (299.xx), specific delays in development (315.xx), tic disorder (307.2x) and hyperkinetic syndrome of childhood (314.xx). In addition, the following medical condition outcomes that are not biologically plausibly linked to postnatal organic-Hg exposure from TCVs were examined: febrile seizure (780.3), failure to thrive (783.4) and cerebral degeneration (330.xx or 331.xx). When there were multiple instances of the same diagnosis in a child, only the first instance was counted. Additionally, to allow for a potential cause and effect relationship between exposure and outcome, only individuals diagnosed with the outcomes examined following administration of the vaccines under study were included in the present analyses as cases. Table 1 summarizes the overall demographics of the cases examined.

**Table 1:** A summary of various types of cases and controls examined in the present study.

Group Examined (ICD-9 Code)	M	F	M/F Ratio	Birth Years	Mean Age of Initial Diagnosis ± SD
**Neurodevelopmental Disorders:**					
Pervasive Developmental Disorder Cases (299.xx)	399	93	4.3	1991–1999	4.1 ± 1.6
Controls	13,482	12,885	1.05	1991–1993	---
Specific Developmental Delay Cases (315.xx)	3916	1783	2.2	1991–2000	2.6 ± 1.6
Controls	24,612	23,915	1.03	1991–1995	---
Tic Disorder Cases (307.2x)	253	91	2.2	1991–1999	5.1 ± 2.0
Controls ^1^	14,327	13,689	1.03	1991–1993	---
Hyperkinetic Syndrome of Childhood Cases (314.xx)	1158	327	3.5	1991–1998	5.7 ± 1.8
Controls ^1^	10,303	10,281	1.00	1991–1993	---
**Non-thimerosal Related Disorders ^2^:**					
Febrile Seizure Cases (780.3)	2425	1784	1.36	1991–2000	1.54 ± 1.29
Controls	43,615	41,496	1.05	1991–1996	---
Failure to Thrive Cases (783.4)	2151	1749	1.23	1991–2000	1.1 ± 0.015 ^3^
Controls	48,780	46,468	1.05	1991–1997	---
Cerebral Degeneration Cases (330.xx or 331.xx)	359	288	1.25	1991–2000	0.63 ± 0.039 ^3^
Controls	69,426	66,462	1.05	1991–1998	---

F: females; ICD-9: International Classification of Disease, 9th revision; M: males; SD: standard deviation; ^1^ These controls were followed to the mean age of initial diagnosis plus one standard deviation; ^2^ These outcomes were specifically chosen as not biologically plausibly linked to postnatal organic-Hg exposure from thimerosal-containing vaccines; ^3^ mean ± standard error.

### 2.4. Determining Controls

To identify controls, who would have only a minimal chance of receiving a diagnosis, controls had to have been continuously enrolled from birth until the mean age of initial diagnosis of the specific diagnosis being assessed plus twice the standard deviation of the mean age of initial diagnosis of that specific diagnosis. The only exceptions were for the specific diagnoses of tic disorder and hyperkinetic syndrome of childhood, where controls had to have been continuously enrolled from birth until the mean age of initial diagnosis of the specific diagnosis being assessed plus the standard deviation of the mean age of initial diagnosis of that specific diagnosis. The length of follow-up was shortened for those two outcomes, because the mean ages of initial diagnosis for tic disorder (5.1 years-old) and hyperkinetic syndrome of childhood (5.7 years-old) were so long that, because of the time limitations on the years of records that were available for study, requiring continuous enrollment from birth until the mean age of initial diagnosis of the specific diagnosis being assessed plus twice the standard deviation of mean age of initial diagnosis would have resulted in virtually no controls available for comparison to those cases. Table 1 summarizes the demographics of the controls examined.

### 2.5. HBV Exposure

The vaccine file for cases and controls was then reviewed to determine the exact dates of HBV administration. Those cases and controls receiving no HBVs were also included in the present study. Overall, for cases and the controls, Hg exposure was assigned as 12.5 µg organic-Hg per dose for those receiving a pediatric HBV or 0 µg organic-Hg per dose for those receiving combined haemophilus influenzae type b (Hib)-HBV or for those receiving neither of the aforementioned vaccines.

### 2.6. Statistical Analyses

The StatsDirect (version 2.8.0) software was utilized for statistical analyses, and a two-sided *p*-value <0.05 was considered statistically significant. The logistic regression function was employed for each of the case *versus* control comparisons examined to determine the odds ratio (OR) per µg organic-Hg from T-HBVs administered within the first 6 months of life. Additionally, the data were separated by gender, and the logistic regression function was employed for each of the case *versus* control comparisons examined to determine the OR per µg organic-Hg from T-HBVs administered within the first 6 months of life. Furthermore, the Fisher’s exact statistical test was utilized for each of the ND cases *versus* control comparisons examined to determine the discrete OR for exposure to 12.5 µg organic-Hg, 25 µg organic-Hg or 37.5 µg organic-Hg from T-HBVs in comparison to 0 µg organic-Hg from HBV or no HBVs administered within the first 6 months of life. The overall null hypotheses for each comparison in the present study was that there would be no difference in the frequency of exposure per µg organic-Hg from T-HBVs between cases and the controls for each of the outcomes examined.

## 3. Results and Discussion

Table 2 displays the relationship between the cases and controls being administered increasing doses of organic-Hg from T-HBVs within the first 6 months of life. On a per µg organic-Hg exposure basis, cases diagnosed with pervasive developmental disorder (OR = 1.054, *p* < 0.001), specific developmental delay (OR = 1.035, *p* < 0.001), tic disorder (OR = 1.034, *p* < 0.001) or hyperkinetic syndrome of childhood (OR = 1.05, *p* < 0.001) were significantly more likely than controls to receive increased organic-Hg exposure from T-HBVs administered within the first 6 months of life. Overall, it was observed for 37.5 µg organic-Hg exposure (the maximum cumulative dose of organic-Hg examined) that cases diagnosed with pervasive developmental disorder (OR = 3.0, 95% confidence interval = 2.3–3.8), specific developmental delay (OR = 2.3, 95% confidence interval = 2.1–2.5), tic disorder (OR = 2.2, 95% confidence interval = 1.5–3.1) or hyperkinetic syndrome of childhood (OR = 2.9, 95% confidence interval = 2.5–3.2) were significantly more likely than controls to receive increased organic-Hg exposure from T-HBVs administered within the first six months of life.

**Table 2:** A summary of exposure to organic-Hg from thimerosal-containing hepatitis B vaccine administration within the first six months of life for the cases diagnosed with a studied neurodevelopmental disorder (ND) and controls.

Group Examined (ICD-9 Code)	0 µg Organic-Hg (%) (Reference Dose)	12.5 µg Organic-Hg (%)	25 µg Organic-Hg (%)	37.5 µg Organic-Hg (%)	Odds Ratio per µg Organic-Hg (95% CI) (*p*-Value)
Pervasive Developmental Disorder Cases (299.xx)	9 (1.8)	4 (0.8)	432 (87.8)	47 (9.6)	1.054 (1.034–1.074) (<0.001)
Controls	1107 (4.2)	533 (2.0)	23,590 (89.5)	1137 (4.3)	
Specific Developmental Delay Cases (315.xx)	115 (2.0)	43 (0.8)	4,878 (85.6)	663 (11.6)	1.035 (1.03–1.04) (<0.001)
Controls	1807 (3.7)	710 (1.5)	42,620 (87.8)	3391 (7)	
Tic Disorder Cases (307.2x)	10 (2.9)	5 (1.5)	296 (86.0)	33 (9.6)	1.034 (1.013–1.057) (<0.001)
Controls ^1^	1163 (4.1)	547 (2)	25,014 (89.3)	1292 (4.6)	
Hyperkinetic Syndrome of Childhood Cases (314.xx)	34 (2.3)	14 (1)	1325 (89.2)	112 (7.5)	1.05 (1.04–1.06) (<0.001)
Controls ^1^	994 (4.8)	469 (2.3)	18,396 (89.4)	725 (3.5)	

Hg: mercury; µg: microgram; ND: neurodevelopmental disorder; ^1^ these controls were followed to the mean age of initial diagnosis plus one standard deviation.

Table 3 displays the relationship between male cases in comparison with male controls and female cases in comparison with female controls being administered increasing doses of organic-Hg from T-HBVs within the first six months of life. On a per µg organic-Hg exposure basis, male cases diagnosed with pervasive developmental disorder (OR = 1.07, *p* < 0.001), specific developmental delay (OR = 1.04, *p* < 0.001), tic disorder (OR = 1.03, *p* < 0.05) or hyperkinetic syndrome of childhood (OR = 1.05, *p* < 0.001) were significantly more likely than male controls to receive increased organic-Hg exposure from T-HBVs administered within the first six months of life. Overall, it was observed for 37.5 µg organic-Hg exposure (the maximum cumulative dose of organic-Hg examined) that male cases diagnosed with pervasive developmental disorder (OR = 3.6, 95% confidence interval = 2.9–4.4), specific developmental delay (OR = 2.5, 95% confidence interval = 2.1–2.9), tic disorder (OR = 2.1, 95% confidence interval = 1.15–2.9) or hyperkinetic syndrome of childhood (OR = 2.9, 95% confidence interval = 2.5–3.2) were significantly more likely than male controls to receive increased organic-Hg exposure from T-HBVs administered within the first six months of life. On a per µg organic-Hg exposure basis, female cases diagnosed with specific developmental delay (OR = 1.03, *p* < 0.001), tic disorder (OR = 1.05, *p* < 0.05) or hyperkinetic syndrome of childhood (OR = 1.04, *p* < 0.001) were significantly more likely than controls to receive increased organic-Hg exposure from T-HBVs administered within the first six months of life. Overall, it was observed for 37.5 µg organic-Hg exposure (the maximum cumulative dose of organic-Hg examined) that female cases diagnosed with specific developmental delay (OR = 2.1, 95% confidence interval = 1.7–2.5), tic disorder (OR = 2.9, 95% confidence interval = 1.4–4.7) or hyperkinetic syndrome of childhood (OR = 2.5, 95% confidence interval = 1.7–3.2) were significantly more likely than female controls to receive increased organic-Hg exposure from T-HBVs administered within the first six months of life.

**Table 3:** A summary of exposure to organic-Hg from thimerosal-containing hepatitis B vaccine administration within the first six months of life when separating male and female ND cases and controls.

Group Examined (ICD-9 Code)	Gender	Odds Ratio per µg organic-Hg (95% CI) (*p*-value)
Pervasive Developmental Disorder Cases (299.xx)	Males	1.07 (1.05–1.09) (<0.001)
	Females	1.01 (0.97–1.05) (>0.56)
Specific Developmental Delay Cases (315.xx)	Males	1.04 (1.03–1.05) (<0.001)
	Females	1.03 (1.02–1.04) (<0.001)
Tic Disorder Cases (307.2x)	Males	1.03 (1.004–1.05) (<0.05)
	Females	1.05 (1.01–1.10) (<0.05)
Hyperkinetic Syndrome of Childhood Cases (314.xx)	Males	1.05 (1.04–1.06) (<0.001)
	Females	1.04 (1.02–1.06) (<0.001)

Hg: mercury; µg, microgram; ND: neurodevelopmental disorder.

By contrast, as summarized in Table 4, for the “not biologically plausibly linked to postnatal organic-Hg exposure from TCVs” outcomes of febrile seizures (OR = 1, *p* > 0.50), failure to thrive (OR = 0.98, *p* < 0.001) or cerebral degenerations (OR = 0.95, *p* < 0.001), the cases were no more likely than the controls to have received increased organic-Hg exposure from T-HBV administered within the first six months of life.

Table 5 summarizes, for each of the ND outcomes examined and controls, the discrete-point ORs (reference exposure = 0 µg organic-Hg) and logistic regression OR estimates for 12.5 µg organic-Hg, 25 µg organic-Hg or 37.5 µg organic-Hg exposure from T-HBVs administered within the first six months of life. Both methods of analysis yielded similar order-of-magnitude results.

**Table 4:** A summary of exposure to organic-Hg from thimerosal-containing hepatitis B vaccine administration within the first six months of life between the cases diagnosed with medical conditions *a priori* not biologically plausibly linked to postnatal organic-Hg exposure from thimerosal-containing vaccines and the controls.

Group Examined (ICD-9 Code)	0 µg Organic-Hg (%) (Reference Dose)	12.5 µg Organic-Hg (%)	25 µg Organic-Hg (%)	37.5 µg Organic-Hg (%)	Odds Ratio per µg Organic-Hg (95% CI) (*p*-value)
Febrile Seizure Cases (780.3)	218 (5.2)	60 (1.4)	3432 (81.5)	499 (11.9)	1 (0.993–1.003) (0.53)
Controls	2858 (3.4)	981 (1.1)	74,102 (87.1)	7,171 (8.4)	
Failure to Thrive Cases (783.4)	322 (8.3)	106 (2.7)	3028 (77.6)	444 (11.4)	0.98 (0.97–0.98) (<0.001)
Controls	3270 (3.4)	1096 (1.2)	82,425 (86.5)	8458 (8.9)	
Cerebral Degeneration Cases (330.xx or 331.xx)	92 (14.2)	13 (2.0)	483 (74.7)	59 (9.1)	0.95 (0.94–0.96) (<0.001)
Controls	5148 (3.8)	1558 (1.1)	116,038 (85.4)	13,145 (9.7)	

Hg: mercury; µg: microgram.

**Table 5:** A summary of exposure to organic-Hg from thimerosal-containing hepatitis B vaccine administration within the first six months of life for the cases diagnosed with a studied ND and the controls by both the discrete-point odds ratio estimates and the logistic regression odds ratio estimates.

Group Examined (ICD-9 Code)	Statistical Approach Used	12.5 µg Organic-Hg (95% CI)	25 µg Organic-Hg (95% CI)	37.5 µg Organic-Hg (95% CI)
Pervasive Developmental Disorder Cases (299.xx)	Discrete Point Odds Ratio	0.92 (0.21–3.3)	2.3 (1.2–5)	5.1 (2.5–10.4)
	Logistic Regression Odds Ratio Estimate	1.7 (1.4–2)	2.3 (1.8–2.8)	3.0 (2.2–3.8)
Specific Developmental Delay Cases (315.xx)	Discrete Point Odds Ratio	0.95 (0.65–1.4)	1.8 (1.5–2.2)	3.1 (2.5–3.8)
	Logistic Regression Odds Ratio Estimate	1.44 (1.4–1.5)	1.88 (1.75–2)	2.3 (2.1–2.5)
Tic Disorder Cases (307.2x)	Discrete Point Odds Ratio	1.1 (0.28–3.4)	1.4 (0.74–2.9)	3 (1.4–6.8)
	Logistic Regression Odds Ratio Estimate	1.4 (1.2–1.7)	1.8 (1.3–2.4)	2.3 (1.5–3.1)
Hyperkinetic Syndrome of Childhood Cases (314.xx)	Discrete Point Odds Ratio	0.87 (0.43–1.7)	2.1 (1.5–3)	4.5 (3.0–6.9)
	Logistic Regression Odds Ratio Estimate	1.6 (1.5–1.7)	2.2 (2–2.5)	2.9 (2.5–3.2)

Hg: mercury; µg: microgram.

Investigators from the CDC and FDA previously described that products, such as vaccines, which are intended for healthy people, must be held to a high standard of safety assurance [8,9,10]. However, the study of vaccine risks is more complex than for therapeutic products, because the exposure is virtually universal for many vaccines, ensuring the chance occurrence of many adverse outcomes in temporal association with vaccines. As a result, these investigators described using the VSD to more rigorously evaluate vaccine-associated risks (hypothesis testing) [8,9,10].

The biological plausibility of the results observed in the present study are supported by previous investigators observing that TCV administration to human infants resulted in some infants having blood and hair Hg levels in excess of safety limits established by the U.S. Environmental Protection Agency (EPA) [11,12,13,14]. In addition, the evaluation of the distribution of Hg following the administration of TCVs to infant monkeys mimicking the U.S. vaccine schedule of the 1990s showed that significant Hg levels were present in the brain [15] and resulted in alterations in the number of neurons in the dentate gyrus of the hippocampus and thalamus [16]. Recently, thimerosal and ethyl-Hg species were shown to be actively transported across neuronal cellular membranes [17,18]. It is of great concern that administration of TCVs results in increased brain Hg concentrations, because once Hg enters the brain, it has a plethora of adverse neurodevelopmental effects associated with the ND outcomes examined in the present study [19].

In further support of the findings of the present study, previous studies showed significant dose-dependent relationships between TCV administration mimicking the childhood vaccine schedule and ND-associated pathology or clinical symptoms in animal model systems [1]. Furthermore, investigators previously observed significant associations between TCV administration to children and NDs using several epidemiological methods in various databases [20,21,22,23,24,25,26]. Finally, a recent meta-analysis reported that significant associations were observed for environmental Hg exposure with autism spectrum disorder (OR = 1.66, 95% confidence interval = 1.14–2.17) and attention-deficit hyperactivity disorder (OR = 1.60. 95% confidence interval = 1.10–2.33) [27].

The results of the present study differ from several other studies that failed to find a consistent significant relationship between ND and organic-Hg exposure from TCVs [28,29,30,31]. This may have occurred, in part, because other studies examined cohorts with significantly different childhood vaccine schedules and with different diagnostic criteria for outcomes. This difference may have also occurred because these other studies employed inappropriate epidemiological methods, especially with respect to the critical issue of follow-up period for individuals examined [22,32].

### Strengths/Limitations

A strength of the present VSD study is that a retrospective assessment of prospectively collected automated medical records of patients HMO-enrolled was undertaken. All cases were HMO-enrolled continuously from birth until a medical diagnosis of an ND was made. Controls were HMO-enrolled continuously from birth for a sufficient time period to ensure that there was a small chance that, during additional follow-up, any of the controls would be medically diagnosed with an ND. As a consequence of the study design employed, all subjects examined were followed on a prospective longitudinal basis. No potential biases as to various independent variables associated with the eventual diagnostic status of subjects would have impacted HMO-enrollment, since all subjects were enrolled from birth and the outcomes examined were diagnosed many months or many years after birth. In addition, differences in healthcare availability among cases and controls were minimized, because all subjects were HMO-enrolled from birth, so healthcare was continuously available for cases/controls.

An additional strength of the present study is that it was decided, *a priori*, to ensure adequate amounts of data for our analyses, that the included controls would be HMO-enrolled continuously from birth until they were at least a mean age of initial diagnosis of ND plus twice the standard deviation of the mean age of initial diagnosis of ND (with the exception of tic disorder and hyperkinetic syndrome of childhood, where, because of VSD records-access limitations, controls were continuously enrolled from in the VSD until they were at least a mean age of initial diagnosis of an ND plus the standard deviation of mean age of initial diagnosis of ND). Based on the data for age of initial diagnosis for an ND, the mean plus twice the standard deviation was a sufficient period to ensure that, with further follow-up, those controls without an ND would probably not receive ND diagnoses in the VSD (mathematically, there is a <2.5% error). For ND outcomes of tic disorder and hyperkinetic syndrome of childhood, where the controls’ were enrolled to the mean plus the standard deviation, there was still a sufficient period to ensure that with further follow-up only slightly more controls without an ND diagnosis would subsequently receive an ND diagnosis (mathematically, there is a <16% error). As shown in Table 6, it was observed by reducing the length of follow-up of controls for the various ND diagnoses examined to only the mean age of initial diagnosis of ND (*i.e.*, cases/controls were followed for the same length of time, and mathematically, there is about a 50% error), the overall adverse effects observed on a per µg basis from increasing exposure to organic-Hg exposure from T-HBV administered within the first six months of life were still statistically significant for most ND outcomes, but the ORs were reduced by about 50%, as would be expected from a normal curve distribution. Clearly, these analyses establish the importance of following controls in any study of NDs for a sufficient period of time to ensure that the risk of a control being subsequently diagnosed with a “case” condition is as small as possible, subject to the limitations of the records that are accessible for study.

Another study strength was that the VSD data were collected independently of the study design used. The VSD data records analyzed were collected as part of the routine healthcare individuals received as part of participation with their respective HMOs, and as such, the healthcare providers in no way were thinking about the potential association between vaccine exposures and potential health outcomes.

A further strength of the present study was that the specific methods employed to evaluate differences in cumulative doses of organic-Hg received at specific intervals during the infant period were based upon the wide-ranging recommendations for routine HBV administration and not the result of a small group of children receiving anomalous exposures to vaccines. This is evidenced by the fact that in 1991, the Advisory Committee on Immunization Practices (ACIP) recommended that infants receive their HBVs as follows: first dose between birth and two months of age, second dose between one and four months of age and third dose between six and 18 months of age [33].

**Table 6:** A summary of the effect of inadequate follow-up of controls on the risk of exposure to organic-Hg from thimerosal-containing hepatitis B vaccine administration within the first six months of life for the cases diagnosed with a studied ND and the reduced follow-up controls ^1^.

Group Examined (ICD-9 Code)	0 µg Organic-Hg (%) (Reference Dose)	12.5 µg Organic-Hg (%)	25 µg Organic-Hg (%)	37.5 µg Organic-Hg (%)	Odds Ratio per µg Organic-Hg (95% CI) (*p*-Value)
Pervasive Developmental Disorder Cases (299.xx)	9 (1.8)	4 (0.8)	432 (87.8)	47 (9.6)	1.02 (1.001–1.03) (<0.05)
Controls	2956 (3.3)	1021 (1.2)	77,092 (87)	7538 (8.5)	
Specific Developmental Delay Cases (315.xx)	115 (2.0)	43 (0.8)	4,878 (85.6)	663 (11.6)	1.021 (1.016–1.025) (<0.001)
Controls	5026 (3.8)	1517 (1.2)	111,969 (85.4)	12,619 (9.6)	
Tic Cases (307.2x)	10 (2.9)	5 (1.5)	296 (86.0)	33 (9.6)	1.01 (0.99–1.03) (>0.30)
Controls	2156 (3.3)	818 (1.3)	56,377 (87.6)	5024 (7.8)	
Hyperkinetic Syndrome of Childhood Cases (314.xx)	34 (2.3)	14 (1)	1325 (89.2)	112 (7.5)	1.01 (1.004–1.02) (<0.01)
Controls	1821 (3.6)	711 (1.4)	44,074 (87.8)	3594 (7.2)	

Hg: mercury; µg: microgram; ^1^ these controls were followed to the mean age of initial diagnosis.

However, the results of the present study may have a number of potential limitations. The results observed may have occurred from unknown biases or cofounders present in the datasets examined. To assess this possibility, other control outcomes (*i.e.*, outcomes that are not biologically plausibly linked to postnatal organic-Hg exposure from TCVs) were examined, such as a diagnosis of failure to thrive, cerebral degenerations or febrile seizures, using the same methodology employed for ND. Since no similar patterns of significant positive dose-dependent associations were observed as that found for organic-Hg exposure from T-HBV administration and the subsequent risk of ND diagnosis, it would seem that there is little risk that there are unknown biases or confounders in the VSD datasets examined.

Additionally, a potential limitation of the present study is that there are differences in the general health status among the individuals examined independent of exposure to organic-Hg from T-HBV administration. It was not only observed that control outcomes that were not biologically plausibly linked to postnatal organic-Hg exposure from TCVs showed no significant positive dose-dependent associations, but it was actually observed that there were significant negative dose-dependent associations for the outcomes of failure to thrive and cerebral degenerations, as shown in Table 4. Furthermore, as shown in Table 5, for most of the ND outcomes examined, the discrete point estimate ORs were less than one for 12.5 µg organic-Hg exposure among cases compared to controls. Overall, these phenomena suggest that a healthy vaccine effect was present in our data. As described previously by investigators from the CDC [34], confounding of this sort is a general problem for studies of adverse reactions to prophylactic interventions, as they may be withheld from some individuals precisely because they are already at high risk of the adverse event, and as a consequence, studies that fail to control adequately for such confounding factors are likely to underestimate the risk of adverse events attributable to vaccination. Since this phenomenon is apparent in the data examined, the significantly increased positive dose-dependent associations observed for the ND outcomes examined are probably underestimates of the true extent of the relationship between organic-Hg exposure from the TCVs and ND outcomes studied.

Another potential limitation is that the results observed may be the result of statistical chance. However, this is unlikely, given the limited number of statistical tests performed, the highly significant results observed (*p*-values observed were <0.001) and the consistency in the direction and magnitude of the results observed.

Other potential limitations include the possibilities that some individuals in the VSD database examined may have had more subtle neurological dysfunction that was not brought to the attention of their healthcare providers, healthcare providers may have misdiagnosed some individuals or some vaccine exposures may not have been appropriately classified. These limitations, while possibly present in the present data examined, should not have significantly impacted the results observed, because it is unclear how differential application would have occurred to produce the results observed. Moreover, misclassification occurring in the data examined would tend to bias any results observed toward the null hypothesis, since such effects would result in individuals being placed in the wrong exposure and/or outcome categories and result in decreased statistical power to determine true potential exposure-outcome relationships.

Another potential limitation is that exposures to other sources of Hg were not evaluated. It is very likely that among the individuals examined in the present study, they incurred other organic-Hg exposures from other TCVs, dental amalgams, fish or other environmental sources. While these other sources of Hg may play a significant involvement in the pathogenesis of NDs, these unaccounted for Hg exposures would actually tend to bias the results observed towards the null hypothesis, because they potentially would confound the specific exposure classifications of Hg examined. For example, individuals classified as having lower organic-Hg exposure from TCVs may have actually received high doses of Hg from other sources, and individuals having higher organic-Hg exposure from TCVs may have actually received low doses of Hg from other sources, with the net result tending to minimize the magnitude of the associations observed.

Finally, the current study suffers from the potential limitation that analyses were not conducted to further explore the precise timing and cumulative doses of organic-Hg from all TCVs associated with maximum adverse consequences. In future studies, it would be worthwhile to further explore these precise-timing and cumulative-doses phenomena. In addition, it would be valuable to evaluate other NDs, as well as other covariates, such as race, birth weight, parental mental illness, socioeconomic status, genetic susceptibility, *etc.*, which may further affect the magnitude of the adverse effects found. This may be particularly true for covariates, such as socioeconomic status, maternal mental illness and genetic susceptibility, which have been shown to play an important role in NDs. It would also be worthwhile to evaluate what, if any, impact matching cases and controls on year of birth would have on the results observed.

## 4. Conclusions

This study provides new epidemiological evidence supporting a significant relationship between increasing organic-Hg exposure from TCVs and the subsequent risk of an ND diagnosis. Future studies should be completed to further evaluate the relationship between other sources of organic-Hg exposure from TCVs and other chronic disorders and to further explore potential subpopulations and the timing of exposure to organic-Hg from TCV administration associated with adverse outcomes. Routine childhood vaccination is an important public health tool to reduce the morbidity and mortality associated with infectious diseases [35]. However, it is also a public health imperative to end the unnecessary addition of thimerosal to vaccines based on data showing an association between its administration and adverse outcomes.
